# Studyholism Inventory (SI-10): A Short Instrument for Evaluating Study Obsession Within the Heavy Study Investment Framework

**DOI:** 10.5964/ejop.v16i4.1911

**Published:** 2020-11-27

**Authors:** Yura Loscalzo, Marco Giannini

**Affiliations:** a Department of Health Sciences, School of Psychology, University of Florence, Florence, Italy; University of Wroclaw, Wroclaw, Poland

**Keywords:** obsession, heavy work investment, study addiction, study engagement, work addiction, workaholism

## Abstract

Loscalzo and Giannini (Loscalzo, Y., & Giannini, M. [2017]. Studyholism or Study Addiction? A comprehensive model for a possible new clinical condition. In A. M. Columbus (Ed.), Advances in psychological research, (Vol. 125, pp. 19-37). Hauppauge, NY, USA: Nova Science) recently proposed a theoretical model for a new potential clinical condition: Studyholism, or obsession toward studying. This study aims to analyze the psychometric properties of the instrument that has been created based on their theory, namely the Studyholism Inventory (SI-10). The participants are 1296 Italian college students aged between 19 and 55 years. We analyzed its factor structure, as well as its convergent and divergent validity, and we proposed the cut-off scores of the SI-10. Moreover, we investigated some demographic and study-related differences in studyholism and study engagement and the correlations with academic indicators. The results showed that the SI-10 is a ten-item (2 fillers) and 2-factor instrument (GFI = .98, CFI = .97, RMSEA = .07) with good psychometric properties. The SI-10 could be used in future research to analyze the features and correlates of studyholism, and for both clinical and preventive purposes, pointing to favor students’ well-being and academic success.

In literature, scholars recently argued about the existence of a new potential clinical condition. It is associated with problematic overstudying, and it has been defined as study addiction (Atroszko, Andreassen, Griffiths, & Pallesen, 2015) or studyholism (i.e., obsession toward studying; Loscalzo & Giannini, 2017a). Hence, this new potential clinical disorder has been conceptualized referring to two different theoretical frameworks, with implications for preventive and clinical interventions. Adopting different theorizations might lead to use strategies that have been validated for diverse clinical diagnoses. By adopting the study addiction definition, the psychologists will probably look for effective strategies used for behavioral addictions or other externalizing disorders. Instead, using the studyholism definition, the psychologists will look for interventions that have been proved effective for the obsessive-compulsive disorder or other internalizing disorders. Therefore, it is critical to shed light on the proper operationalization of problematic overstudying through studies using instruments that allow evaluating both study addiction and studyholism and that have good psychometric properties.

There are currently the Polish and Norwegian versions of the Bergen Study Addiction Scale (BStAS; Atroszko et al., 2015), and its Italian version that, however, showed some psychometric issues (Loscalzo & Giannini, 2018a). Moreover, there are also available the Polish and Italian versions of the Studyholism Inventory (SI-10; Loscalzo, Giannini, & Golonka, 2018) and the Croatian, Spanish, and English translations. Though, Loscalzo et al. (2018) presented the SI-10 preliminary analyses only. Concerning the Italian SI-10, the paper reports just the results of the Exploratory Factor Analysis (EFA) performed by the authors for reducing their initial pool of 68 items. Hence, the present study aims to deepen the psychometric analysis of the SI-10 in Italian college students. Next, the SI-10 (as well as the BStAS) might be used to deepen the analysis of problematic overstudying to prevent this construct from the issues that characterize workaholism, or the clinical condition associated with problematic overworking. Indeed, since the 1970s, when Oates (1971) coined the term workaholism, many studies have been conducted on this psychological condition, often using different definitions of the construct. Therefore, despite the massive literature about this topic, there is not a consensus about the definition of workaholism, neither about its internalizing or externalizing nature (Giannini & Loscalzo, 2016; Loscalzo & Giannini, 2015, 2017b). Thus, it is not surprising that Andreassen, Hetland, and Pallesen (2014) did not find adequate convergent validity among three of the most used instruments for evaluating workaholism.

As written above, presently, there are two different conceptualizations of problematic overstudying. However, Loscalzo and Giannini (2017a) stressed that there are some issues concerning both the definition of study addiction (Atroszko et al., 2015) and the instrument used for its evaluation, that is the BStAS (Atroszko et al., 2015). Study addiction is defined as a behavioral addiction characterized by the seven core components of addictions (i.e., salience, tolerance, mood modification, relapse, withdrawal, conflict, and problems). To evaluate it, Atroszko et al. (2015) proposed the BStAS, which they defined as an adaptation of the Bergen Work Addiction Scale (BWAS; Andreassen, Griffiths, Hetland, & Pallesen, 2012). More specifically, they changed the word “work” with “study” in the BWAS items. However, as Korb (2012) outlined, adapting an instrument requires adding, removing, and/or significantly varying the content of the items of the original one. Hence, as they merely changed the behavior-related word in each of the BWAS items, they did not create a new instrument in its strict meaning. In addition, the Italian version of the BStAS (Loscalzo & Giannini, 2018a) does not have fully satisfying psychometric properties. Moreover, Loscalzo and Giannini (2017a) stressed that, as suggested by Kardefelt-Winther (2015), when studying a new potential behavioral addiction we should go beyond the a priori assumption that it is an addiction, for detecting the real manifestation of the problem behavior. It seems even more critical in the analysis of problematic overstudying since, by proposing some similarities with workaholism, we should keep in mind that workaholism itself still lacks a shared definition and operationalization by the scientific community. For this reason, Loscalzo and Giannini (2017a), in line with the suggestion of Quinones and Griffiths (2015), developed a definition and a comprehensive model for the study of problematic overstudying before creating a new instrument to evaluate it, instead of simply proposing an analogy between workaholism and studyholism.

Loscalzo and Giannini (2017a, 2018b) proposed to define this new potential clinical condition “studyholism” since this term, not including the word “addiction,” avoids reducing it to externalizing (or addiction) symptoms and to negative aspects only. After some preliminary analyses, Loscalzo and Giannini (2017a) defined (clinical) studyholism as “a possible new clinical condition which is characterized by internalizing symptoms (i.e., obsessive-compulsive symptoms such as constant thinking to study or inner drive to study) and by low levels of study engagement” (p. 31). They stressed indeed that studyholism might be associated with either low (disengaged studyholism) or high (engaged studyholism) levels of study engagement, which is a study-related positive feature that is characterized by vigor, dedication, and absorption (Schaufeli, Salanova, González-Romá, & Bakker, 2002) and that, for Loscalzo and Giannini (2017a), includes inner motivation for studying. More specifically, study engagement is positively associated with well-being (e.g., Cadime et al., 2016; Li & Lerner, 2011; Wang, Chow, Hofkens, & Salmela-Aro, 2015) and academic success (e.g., Chase, Hilliard, Geldhof, Warren, & Lerner, 2014; Salmela-Aro, Kiuru, Leskinen, & Nurmi, 2009).

Loscalzo and Giannini (2017a) underlined that it is valuable to analyze studyholism referring to the Heavy Study Investment framework (see Snir & Harpaz [2012] for the heavy work investment model). By distinguishing among disengaged studyholics, engaged studyholics, and engaged students, it helps avoid overpathologizing a common behavior such as studying (Billieux, Schimmenti, Khazaal, Maurage, & Heeren, 2015). In brief, crossing studyholism and study engagement, Loscalzo and Giannini (2017a, 2018b) proposed four kinds of student, three of which are heavy study investors. These students are: 1) disengaged studyholics, namely students with high studyholism and low study engagement; 2) engaged studyholics, who are characterized by high levels of both studyholism and study engagement; 3) engaged students, namely students with high study engagement and low studyholism. They are heavy study investors as the two previous kinds of student, but they have not an obsession toward studying; 4) detached students, who have low levels of both studyholism and study engagement. They are low study investors and, even if they are not studyholics, they deserve an intervention since they have not a positive attitude toward the study.

Moreover, Loscalzo and Giannini (2017a) developed a comprehensive model of studyholism that comprehends its antecedents and outcomes, and that they recently tested in many of its suggested variables (Loscalzo & Giannini, 2019a).

Finally, aiming to evaluate studyholism, Loscalzo et al. (2018) settled a pool of 68 items based on Loscalzo and Giannini (2017a)’s first definition of studyholism as a three-factor construct characterized by addiction (or externalizing) symptoms, obsessive-compulsive (or internalizing) symptoms, and high or low study engagement. However, once they administered the pilot version to Italian college students to reduce the number of items, aiming to have a short instrument for quick screening, they found a ten-item and two-factor solution using EFA: 1) Studyholism, which is composed of five items covering obsessive-compulsive symptoms. This factor does not include addiction symptoms and study-related perfectionism, even if the pool of items covered them; 2) Study Engagement, which comprehends five items as well, and that does not comprehend any item related to vigor, dedication, and absorption (the three main components of study/work engagement, as defined by Schaufeli et al., [2002]), even if the pilot version included them. It covers mainly intrinsic motivation toward study, which is a positive study-related dimension as well. Based on these results, the theoretical construct of studyholism has been re-analyzed and defined as a 2-factor construct characterized by obsessive-compulsive symptoms and high or low study engagement (Loscalzo & Giannini, 2017a).

In sum, problematic overstudying seems to be not a pure behavioral addiction (Atroszko et al., 2015) neither a clinical disorder characterized by both addiction and obsessive-compulsive symptoms, as preliminarily hypothesized by Loscalzo and Giannini (2017a). It seems to be better defined as an obsessive-compulsive related disorder (Loscalzo & Giannini, 2017a, 2018b, 2018c, 2019a; Loscalzo et al., 2018). However, further studies are needed to analyze its internalizing and/or externalizing nature.

Hence, this paper aims to present the psychometric properties of the SI-10 (Loscalzo et al., 2018), which may be used in future research to disentangle the open question about its nature and for analyzing its correlates. It may also be used to detect students who need interventions to foster their well-being and academic success. More specifically, we aim to further analyze the psychometric properties of the SI-10 on Italian college students by analyzing its factor structure, internal reliability, and convergent and divergent validity. Moreover, we aim to propose the cut-off scores for defining high and low levels of studyholism/study engagement. These scores may be used to detect the four kinds of student proposed by Loscalzo and Giannini (2017a) in the general population. Finally, we aim to analyze some demographic (e.g., gender) and study-related differences (e.g., major of study) in studyholism and study engagement, and the association between studyholism, study engagement and academic indicators, such as time investment in studying and grade point average (GPA).

## Method

### Participants

We recruited a sample of 1296 Italian college students aged between 19 and 55 years (*M* = 23.15, *SD* = 4.51). They were mostly females (75.5%), and half of them (50.5%) lived in Florence, which is part of Central Italy. A subsample of 340 students was gathered for a previous study to reduce the total number of items of the test (Loscalzo et al., 2018); in the present study, we used it for new analyses.

Table 1 shows the socio-demographic and study-related characteristics of the samples: 1) Sample 1 (*n* = 956), or the “new” sample, on which we performed Confirmatory Factor Analyses (CFAs) and evaluated internal reliability. Also, on a random subsample of 474 students (about 50% of the total sample), we analyzed the convergent and divergent validity of the instrument; 2) Sample 2 (*n* = 340), or the “old” sample, on which Loscalzo et al. (2018) previously performed EFA; and 3) Sample 3 (*N* = 1296), namely the whole sample, on which we calculated the cut-off scores of the SI-10 and performed some group differences analyses.

**Table 1:** Socio-Demographic and Study-Related Characteristics of Participants (N = 1296)

Variable	Sample 1 (*n* = 956)	Sample 2 (*n* = 340)	Sample 3 (*N* = 1296)
Gender			
Males	22.7%	29.4%	24.5%
Females	77.3%	70.6%	75.5%
Age			
Range	19-55	19-54	19-55
*M* (*SD*)	23.70 (4.71)	21.59 (3.46)	23.15 (4.51)
Living in Florence			
Yes	32.9%	100%	50.5%
No	67.1%	0.0%	49.5%
Major of study			
Medical Studies	14.5%	2.4%	11.3%
Help Professions	2.8%	44.1%	13.7%
Psychology	15.8%	32.6%	20.2%
Humanities and Education	29.4%	0.9%	21.9%
Math, Physics, Sciences, and Engineering	21.5%	3.2%	16.7%
Social Sciences (e.g., Law, Economy)	15.6%	16.5%	15.8%
Missing	0.4%	0.3%	0.4%
Year			
First	17.7%	50.6%	26.3%
Second	20.6%	36.8%	24.8%
Third	26.4%	7.6%	21.5%
Fourth	14.2%	2.4%	11.1%
Fifth	21.1%	2.6%	16.3%
Rejected before university and/or late with studies			
Yes	27.9%	19.1%	25.6%
No	72.1%	80.0%	74.2%
Missing	0.0%	0.9%	0.2%
Hours a day of study			
Range	0-16	0-12	0-16
*M* (*SD*)	4.58 (2.22)	3.66 (1.84)	4.34 (2.17)
Days a week of study			
Range	0-7	0-7	0-7
*M* (*SD*)	5.32 (1.27)	5.09 (1.16)	5.26 (1.25)
Hours a day of study before exams			
Range	1-16	1-16	1-16
*M* (*SD*)	7.14 (2.35)	6.52 (2.21)	6.97 (2.33)
Days a week of study before exams			
Range	1-7	1-7	1-7
*M* (*SD*)	6.45 (0.87)	6.30 (0.99)	6.41 (0.90)
Studying on the weekend			
Yes	82.5%	85.9%	83.4%
No	17.5%	13.5%	16.4%
Missing	0.0%	0.6%	0.2%
Grade point average^a^			
Range	19-31	18-30	18-31
*M* (*SD*)	26.56 (2.19)	25.31 (2.06)	26.24 (2.23)
Studyholism, SI-10^b^			
Range	4-20	4-20	4-20
*M* (*SD*)	14.04 (3.77)	12.74 (3.64)	13.70 (3.77)
Study Engagement, SI-10^b^			
Range	4-20	4-20	4-20
*M* (*SD*)	14.62 (3.38)	14.66 (3.08)	14.42 (3.36)

^a^Italian grades range between 18 and 30, we refer to 31 for the grade “30 with laudem”. ^b^SI-10 = Studyholism Inventory; the SI-10 Studyholism and Study Engagement scales are calculated referring to the final version of the SI-10 (i.e., 4 items and 1 filler for each scale).

### Materials

#### Studyholism Inventory (SI-10)

The SI-10 (Loscalzo et al., 2018) is a test comprehending a head-sheet with some questions related to study habits (e.g., hours of study a day generally, usually studying on the weekend) followed by 10 items that allow evaluating Studyholism and Study Engagement through five items per scale. A sample item for the Studyholism scale is “Often, I feel anxious or nervous because of study-related issues,” while for the Study Engagement scale is “My desire to get good grades motivates me to study” (see Appendix for the English back-translation of all the items). The participants have to rate each item through a 5-point Likert scale, ranging between 1 (*Strongly disagree*) and 5 (*Strongly agree*). There are available the English, Polish, Croatian, and Spanish translations of this instrument; in the present research, we administered the Italian 10-item version.

#### Bergen Study Addiction Scale (BStAS)

We administered the Italian version (Loscalzo & Giannini, 2018a) of the BStAS (Atroszko et al., 2015). It is an instrument comprehending seven items to be rated on a 5-point Likert scale (1 = *Never*, 5 = *Always*). More specifically, there is one item for each of the seven core components of substance addictions (i.e., salience, tolerance, mood modification, relapse, withdrawal, conflict, and problems). The instructions require the participants to rate how often during the last year they experienced the situations asked in the items (e.g., having studied so much that this has had a negative influence on their health). The BStAS showed for its one-factor structure a good fit on Norwegian students and an acceptable fit on Polish students; its internal reliability is average in both samples, respectively, .74 and .75 (Atroszko et al., 2015). As far as the Italian version is concerned, Loscalzo and Giannini (2018a) did not find an acceptable fit, indicating items 1 and 2 as the most critical. However, they found satisfactory internal reliability (α = .72). In the present study (*n* = 956), Cronbach’s alpha is of .73.

#### Utrecht Work Engagement Scale – Student and Short Version (UWES-S-9)

We administered the Italian version (Loscalzo & Giannini, 2019b) of the UWES-S-9 (Schaufeli & Bakker, 2004). It is a 9-item instrument that evaluates study engagement in its three components: vigor, dedication, and absorption. An example of item for each of the three scales is, respectively, “When I’m doing my work as a student, I feel bursting with energy,” “I am enthusiastic about my studies,” and “I get carried away when I am studying.” Both the original and the Italian versions showed good psychometric properties: a good fit for the three-factor model and good internal reliability for both the total (respectively, .84 and .90) and the Vigor, Dedication, and Absorption subscales (respectively, .73, .76, .70, and .82, .88, .76). In the present study (*n* = 956), the Cronbach’s alpha values are: total score = .89, Vigor = .80, Dedication = .89, Absorption = .75.

### Procedure

Once we obtained the authorization from the Department of Health Sciences of the University of Florence, we created an online questionnaire containing some personal data, such as age, gender, and city of living, followed by the SI-10 (which comprehends some questions related to study habits), the BStAS, and the UWES-S-9. On the first page of the online questionnaire, we informed participants about our research aims and that, by completing the questionnaire they gave us their informed consent.

### Data Analysis

We conducted the analyses using SPSS (Version 24) and AMOS (Version 22).

First, on Sample 1 (*n* = 956), we conducted some CFAs on the SI-10, aiming to evaluate its factor structure. We used the following indexes and cut-off values to evaluate the fit of the models: χ^2^/df ratio, which indicates a good fit if its value is less than 3 (Byrne, 2001); Goodness of Fit Index (GFI), Comparative Fit Index (CFI), Tucker-Lewis Index (TLI), and Normed Fix Index (NFI), whose cut-off values are: < .90 lack of fit, .90-.95 good fit, > .95 excellent fit (Hu & Bentler, 1999; Kline, 2016); Root mean square error of approximation (RMSEA), for which values below .05 indicate an excellent fit, while values between .05 and .08 indicate an acceptable fit (Kline, 2016; Reeve et al., 2007).

Next, once we established the final version of the SI-10, we calculated the descriptive statistics of the scales included in this study, and their zero-order correlation (*n* = 956); hence, we deepened the analysis of the psychometric properties of the SI-10. First, we calculated the internal reliability (Cronbach’s alpha) of the two subscales (*n* = 956). Also, through Pearson’s correlation, we evaluated its convergent and divergent validity with the UWES-S-9, the BStAS, and the self-reported GPA on a random subsample of participants (*n* = 474).

After that, we calculated on Sample 3 (*N* = 1296) the cut-off scores for defining high and low studyholism and study engagement. More specifically, we calculated the *T* scores for the two scales, and we looked for the raw scores corresponding to -1 *SD* (or 40 *T* score) and +1 *SD* (or 60 *T* score). Next, we calculated the percentage of participants belonging to the four kinds of student that could be detected by the SI-10: disengaged studyholic, engaged studyholic, engaged student, detached student.

Next, we performed six MANOVAs on Sample 3 (*N* = 1296) for analyzing demographic and study-related differences (e.g., gender, year of study) on studyholism and study engagement. In order to control for multiple testing, we applied the Bonferroni correction. Hence, we adjusted the alpha level to .004 (Chen, Feng, & Yi, 2017). If the variable comprehended more than two groups, a Bonferroni post-hoc test was performed. As a preliminary step, we checked on the total sample (*N* = 1296) that Studyholism and Study Engagement scales distributions were compatible with a normal distribution: skewness was respectively -0.28 and -0.47, while kurtosis was -0.69 and -0.10.

Finally, we used Pearson’s correlation for analyzing the association between studyholism and study engagement and age, as well as with some academic indicators evaluated by the SI-10: hours a day of study generally and before exams, and days a week of study generally and before exams (*n* = 474).

## Results

### Psychometric Properties of the SI-10

**Figure 1:**
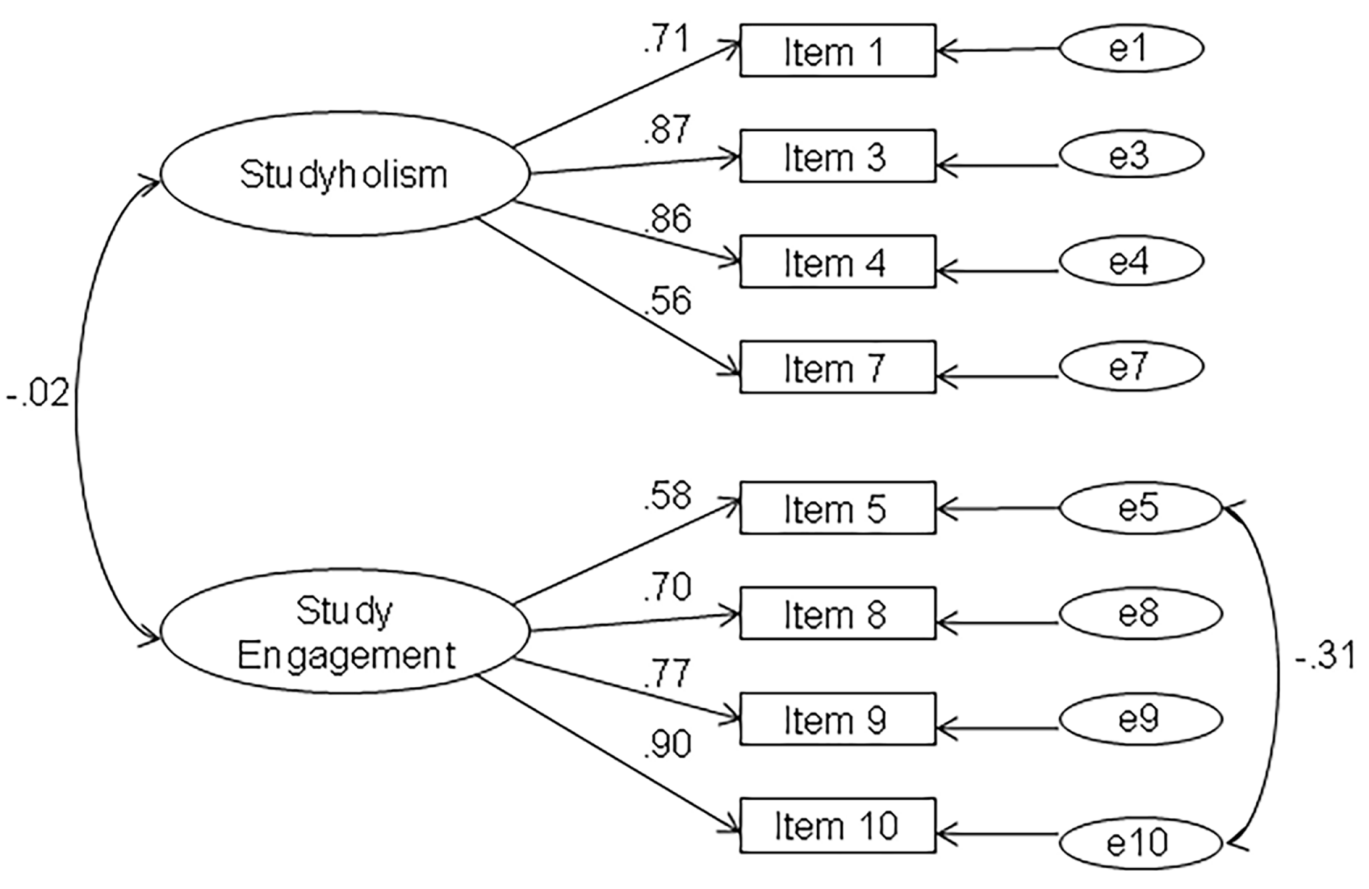
Eight-item and two-factor model, Studyholism Inventory (SI-10; *n* = 956).

First, we conducted a CFA on Sample 1 (*n* = 956) to test if the 2-factor and 10-item model found by Loscalzo et al. (2018) on the Italian sample using EFA fits our data well (Model 1). We did not find a satisfactory fit for this model. For this reason, based on the results of this CFA, we tested another model in which we deleted one item per factor; more specifically, we did not include the ones with the lowest loadings: item 6 for Studyholism and item 2 for Study Engagement (Model 2). This model showed a better fit to the data, which improved by correlating two errors as suggested by modification indices (Model 3). More specifically, the errors correlated are those of the items 5 and 10, and their correlation is theoretically justified, as they are both Study Engagement items. Moreover, the negative error correlation (-.31) is justified by the fact that item 10 is the one that represents the most the factor (as having the highest factor loading). Item 5 is instead the one with the lowest factor loading, and it is the only Study Engagement item not comprehending a reference to “good grades.” Table 2 shows the three models’ fit indexes, while Figure 1 shows the graphical representation of Model 3.

**Table 2:** Fit Indexes for the Three Confirmatory Factor Analyses Models of the Studyholism Inventory (n = 956)

Model	χ^2^/*df*	*p*	GFI	CFI	TLI	NFI	RMSEA
1 – 5 items per factor	18.06	< .001	.89	.86	.81	.85	.13
2 – 4 items per factor	6.52	< .001	.97	.97	.95	.96	.08
3 – Model 2 with one EC	5.74	< .001	.98	.97	.96	.97	.07

*Note.* GFI = Goodness of Fit Index; CFI = Comparative Fit Index; TLI = Tucker-Lewis Index; NFI = Normed Fix Index; RMSEA = Root Mean Square Error of Approximation; EC = Errors’ correlation (correlation between error 5 and error 10).

Once we established the final version of the SI-10, namely ten items and two scales (i.e., Studyholism and Study Engagement), each composed of five items (one filler), we examined further its psychometric properties. As a preliminary step, we analyzed the descriptive statistics of the study variables, and their zero-order correlation (see Table 3).

**Table 3:** Descriptive Statistics and Zero-Order Correlations (n = 956)

Scale	Range	*M* (*SD*)	1	2	3	4	5	6	7	8	9	10	11	12
1. SI-10, Studyholism	4-20	14.04 (3.77)	-											
2. SI-10, Study Engagement	4-20	14.62 (3.38)	-.01	-										
3. BStAS, Study Addiction	7-35	19.51 (5.32)	.58***	.15***	-									
4. UWES-S-9, Total	0-54	33.75 (9.79)	-.18***	.53***	.05	-								
5. UWES-S-9, Vigor	0-18	9.91 (3.74)	-.21***	.39***	-.04	.86***	-							
6. UWES-S-9, Dedication	0-18	13.11 (3.86)	-.23***	.50***	-.03	.86***	.58***	-						
7. UWES-S-9, Absorption	0-18	10.72 (3.76)	-.03	.48***	.19***	.87***	.64***	.62***	-					
8. Grade point average^a^	19-31	26.56 (2.19)	-.15***	.43***	-.08*	.22***	.14***	.24***	.19***	-				
9. Hours/Day generally	0-16	4.58 (2.22)	.17***	.29***	.26***	.20***	.11***	.13***	.29***	.09**	-			
10. Days/Week generally	0-7	5.32 (1.27)	.09**	.22***	.17***	.22***	.14***	.15***	.29***	.05	.48***	-		
11. Hours/Day exams	1-16	7.14 (2.35)	.16***	.22***	.24***	.17***	.09**	.10**	.27***	.11**	.63***	.27***	-	
12. Days/Week exams	1-7	6.45 (0.87)	.12***	.27***	.18***	.15***	.08*	.13***	.17***	.09**	.28***	.46***	.38***	-

*Note.* SI-10 = Studyholism Inventory, Studyholism and Study Engagement scales are calculated referring to the final version of the SI-10 (i.e., 4 items and 1 filler for each scale); BStAS = Bergen Study Addiction Scale; UWES-S-9 = Utrecht Work Engagement Scale, Student and Short Version; Hours/Day generally = Hours a day of study generally; Days/Week generally = Days a week of study generally; Hours/Day exams = Hours a day of study before exams; Days/Week exams = Days a week of study before exams.

^a^Italian grades range between 18 and 30, we refer to 31 for the grade “30 with laudem”.

*p < .02. **p < .01. ***p < .001.

Concerning internal reliability, we found an average Cronbach’s alpha value for both the scales: Studyholism, .84; Study Engagement, .81. Moreover, we evaluated the SI-10 convergent and divergent validity through the correlations between the subscales of the SI-10 and the BStAS and the UWES-S-9, as well as between the SI-10 scales and the self-reported GPA. The correlations have been calculated on a random subsample of Sample 1 (*n* = 474; see Table 4). The results supported the convergent and divergent validity of the SI-10, especially for the correlations with the UWES-S-9 and the GPA: we found positive correlations for the SI-10 Study Engagement scale and negative correlations for the SI-10 Studyholism scale. The BStAS, instead, has a positive correlation with the SI-10 Studyholism scale. Though, it also has a positive, even if low, value of correlation with the SI-10 Study Engagement scale, which however is in line with previous studies about the correlation between study addiction (as evaluated through the BStAS) and study engagement (Atroszko et al., 2015; Loscalzo & Giannini, 2018a).

**Table 4:** Convergent and Divergent Validity of the Studyholism Inventory (SI-10) and Correlation With Academic Indicators (n = 474)

Variable	Studyholism (SI-10)	Study Engagement (SI-10)
Study Addiction (BStAS)	.55***	.18***
Study Engagement – Total (UWES-S-9)	-.19***	.56***
Vigor (UWES-S-9)	-.20***	.43***
Dedication (UWES-S-9)	-.24***	.53***
Absorption (UWES-S-9)	-.05	.49***
Grade point average (GPA)	-.19*	.40***
Hours per day of study generally	.17***	.27***
Days per week of study generally	.10*	.20***
Hours per day of study before exams	.18***	.24***
Days per week of study before exams	.13**	.29***

*Note.* BStAS = Bergen Study Addiction Scale; UWES-S-9 = Utrecht Work Engagement Scale, Student and Short version.

*p = .03. **p = .007. ***p < .001.

### Cut-Off Scores for the Screening of the Four Types of Student

Since we aimed to propose an instrument that allows evaluating studyholism and study engagement, but also to detect four kinds of student (i.e., disengaged studyholics, engaged studyholics, engaged students, detached students) based on high/low levels of studyholism and study engagement, we calculated the cut-off scores for the SI-10 scales.

More specifically, we calculated the *T* scores for the two scales on the total sample (*N* = 1296), and we looked for the raw scores corresponding to -1 *SD* (or 40 *T* score) and +1 *SD* (or 60 *T* score). Based on these analyses, we selected 10 and 18 as the cut-off scores for low and high studyholism, and 11 and 18 for low and high study engagement. Hence, students who score between 4 and 9 on Studyholism have low levels of it, while those who score between 19 and 20 have high studyholism. Concerning study engagement, scores between 4 and 10 indicate low study engagement, while a score between 19 and 20 means high study engagement. See Table 5 for a visual representation of the types of student based on the suggested cut-off scores.

**Table 5:** Profiling of the Four Types of Student by Means of the Studyholism Inventory (SI-10) and Its Cut-Off Scores

Study Engagement score	Studyholism score	
	4-9 (Low)	19-20 (High)
**4-10 (Low)**	Detached student	Disengaged studyholic
**19-20 (High)**	Engaged student	Engaged studyholic

Through the above-mentioned cut-off scores, we found 140 students (10.8%) with high studyholism, 202 (15.6%) with low studyholism, 157 (12.1%) with high study engagement, and 156 (12.1%) with low study engagement. Then, by crossing high and low levels of both studyholism and study engagement, we screened the total sample, and we identified 22 (1.7%) disengaged studyholics, 28 (2.2%) engaged studyholics, 24 (1.8%) engaged students, and 39 (3.0%) detached students.

### Demographic and Study-Related Differences

We conducted six MANOVAs with Studyholism and Study Engagement scales as dependent variables to analyze some demographic and study-related differences (*N* = 1296).

Concerning *gender*, the multivariate tests showed a statistically significant effect, *F*(2, 1293) = 33.55, *p* < .001, partial η^2^ = .05. More specifically, follow-up ANOVAs showed statistically significant differences between males and females on both studyholism and study engagement; respectively: *F*(1, 1294) = 49.15, *p* < .001, partial η^2^ = .04 and *F*(1, 1294) = 19.27, *p* < .001, partial η^2^ = .02. Females have higher levels of both studyholism (*M* = 14.11, *SD* = 3.70) and study engagement (*M =* 14.66, *SD* = 3.30) than males (respectively, *M* = 12.43, *SD* = 3.73 and *M* = 13.71, *SD* = 3.46).

Concerning *doing an activity or a sport besides studying*, we did not find a statistically significant multivariate effect: *F*(2, 1293) = 2.36, *p* = .10, partial η^2^ = .004.

About *usually studying on the weekend* more than once a month, the multivariate test yield statistical significance, *F*(2, 1291) = 20.20, *p* < .001, partial η^2^ = .03. More specifically, who usually study on the weekend have statistically significant higher levels of both studyholism, *F*(1, 1292) = 10.85, *p* = .001, partial η^2^ = .01, and study engagement, *F*(1, 1292) = 30.65, *p* < .001, partial η^2^ = .02, compared to who do not usually study on the weekend. The average score (and *SD*) for those who study and who do not on the weekend are respectively 13.85 (3.71) and 12.92 (3.98) for studyholism, and 14.65 (3.26) and 13.27 (3.65) for study engagement.

In addition, as far as concerns *having repeated a school year before university or being now late with the studies*, we found a statistically significant multivariate effect: *F*(2, 1290) = 16.46, *p* < .001, partial η^2^ = .03. Follow-up ANOVAs showed a difference for both studyholism, *F*(1, 1292) = 8.95, *p* = .003, partial η^2^ = .01, and study engagement, *F*(1, 1292) = 22.30, *p* < .001, partial η^2^ = .02. More specifically, the average (and *SD*) score for who answered yes and no to this question are respectively 14.22 (3.95) and 13.51 (3.70) for studyholism, and 13.68 (3.59) and 14.68 (3.24) for study engagement.

About the *year of study*, the multivariate test showed a statistically significant effect: *F*(8, 2580) = 4.84, *p* < .001, partial η^2^ = .02. However, there is not a statistically significant difference on studyholism, as highlighted by the follow-up ANOVA (using the adjusted alpha level): *F*(4, 1292) = 2.57, *p* = .04, partial η^2^ = .01. Instead, concerning study engagement, the results showed a statistically significant difference, *F*(4, 1291) = 7.08, *p* < .001, partial η^2^ = .02. Post-hoc Bonferroni analysis reported that the fifth year has a statistically significant higher level of study engagement (*M* = 15.40, *SD* = 3.49) than the first (*M* = 14.28, *SD* = 3.23; *p* = .001), the second (*M* = 14.11, *SD* = 3.45; *p* < .001), and the third (*M* = 14.00, *SD* = 3.26; *p* < .001) year, but not than the fourth (*M* = 14.84, *SD* = 3.49) year. No other differences between groups emerged.

Finally, the multivariate test showed a statistically significant effect for the *major of study: F*(10, 2570) = 3.92, *p* < .001, partial η^2^ = .02. More specifically, we found a statistically significant difference for both studyholism, *F*(5, 1286) = 4.14, *p* = .001, partial η^2^ = .02, and study engagement, *F*(5, 1286) = 3.80, *p* = .002, partial η^2^ = .02. The Bonferroni post-hoc test showed that the humanities and educational studies group scores statistically significantly higher (*M* = 14.18, *SD* = 3.77) than both the health professions (*M* = 13.07, *SD* = 3.58; *p* = .032) and the psychology (*M* = 13.07, *SD* = 3.73, *p* = .008) groups on the Studyholism scale. We did not find any other statistically significant difference between medical studies (*M* = 14.19, *SD* = 3.83), math, physics, science and engineering (*M* = 13.76, *SD* = 3.74) and social sciences (*M* = 13.97, *SD* = 3.81) groups. Moreover, the humanities and educational studies group scores also statistically significantly higher (*M* = 14.96, *SD* = 3.25, *p* = .008) than the social sciences group (*M* = 13.89, *SD* = 3.38) on the Study Engagement scale. No other differences between the health professions (*M* = 14.05, *SD* = 3.28), psychology (*M* = 14.56, *SD* = 3.37), medical studies (*M* = 14.84, *SD* = 3.50), math, physic, science and engineering (*M* = 14.14, *SD* = 3.31) groups were detected.

Lastly, we analyzed on a random subsample of Sample 1 (*n* = 474) the correlations between studyholism and study engagement and age, as well as between the following study-related variables: hours of study per day generally and before exams, days of study per week generally and before exams. Table 4 shows the results of these analyses. Concerning age, we did not find a statistically significant relationship with studyholism and study engagement; moreover the *r* values are near zero (i.e., respectively, -.02 and .01).

## Discussion

The present study showed good psychometric properties for the SI-10. Regarding the factor structure, we did not find a good fit for the 10-item version proposed by Loscalzo et al. (2018). However, by deleting one item per each factor (the ones with the lowest factor loadings) and (even more) correlating two errors, we found a good fit. Even if these two items should not be used for scoring purposes, we suggest administering them anyway as they may help the clinician to have more insight into the study-related behaviors of the person. We also found average internal reliability, with values higher than .80 for both the scales of the test. Finally, the results showed good convergent and divergent validity for the SI-10.

The SI-10 Studyholism scale has a positive and statistically significant correlation with the BStAS, which evaluates the same problem behavior (i.e., problematic overstudying) using addiction symptoms instead of obsessive-compulsive ones. The medium value of correlation (.55) indicates that even if they evaluate the same problem behavior, they assess different aspects of it.

Moreover, the Studyholism scale has good divergent validity since, besides the no-significant correlation with the absorption subscale of the UWES-S-9, it has low and negative statistically significant correlations with both the total score and the vigor and dedication subscales (values ranging between .19 and .24). Hence, compared to the Italian version of the BStAS (Loscalzo & Giannini, 2018a), the SI-10 Studyholism scale has better divergent validity.

Concerning the SI-10 Study Engagement scale, it has good convergent validity, as shown by the statistically significant values of correlation with both the total and the three subscales of the UWES-S-9 (values ranging between .43 and .56). As previously said for the correlation between the SI-10 Studyholism scale and the BStAS, these values indicate that the two instruments evaluate the same construct, namely study engagement, but referring to different components of it. We have to keep in mind that the final version of the SI-10 has no item related to vigor, dedication, and absorption (even if initially included in the pool of items for developing the SI-10). It evaluates mainly intrinsic motivation toward studying.

About divergent validity, the SI-10 Study Engagement scale has a statistically significant and positive, even if low, correlation with the BStAS (*r* = .18). However, this is in line with the study of Atroszko et al. (2015) that, using a single item asking students to indicate how much they were engaged in study, found a positive and significant correlation with the BStAS (*r* = .48, *p* < .01). Moreover, this result supports Loscalzo and Giannini’s (2017a) model, which suggested that some studyholics could also be engaged in their study, hence being characterized by a positive aspect too. Also, it seems to support Loscalzo and Giannini’s (2018a) speculation that the Italian BStAS does not adequately distinguish between study addiction and study engagement. In line with this, the results of the zero-order correlations (*n* = 956) showed that the BStAS has a positive correlation with the UWES-S-9 absorption scale. Moreover, the correlations between the BStAS and the time spent studying (i.e., hours per day and days per week of study, both generally and before exams) are higher than those between the SI-10 Studyholism scale and time spent studying. About this, it is interesting to note that also the SI-10 Study Engagement scale has higher correlations with time spent studying than the SI-10 Studyholism scale. Also, the BStAS correlates with three of the four variables related to the time spent studying with values very similar to the ones of the UWES-S-9 absorption scale. In sum, these results seem to provide further support to the assertion that the Italian BStAS does not adequately distinguish between study addiction and study engagement and, more specifically, that it evaluates, in particular, the study engagement component of absorption toward studying.

Finally, concerning the correlations between the SI-10 scales and the GPA, we found a low and negative statistically significant correlation (-.19) between studyholism and GPA. This is in contrast with Atroszko et al. (2015), who found a no-significant value of .02 for the correlation between the BStAS and the GPA of the last semester, and a low positive value of .12 for the GPA of the whole studies. Moreover, the present study showed a very low correlation (*r* = -.08) between the BStAS and the GPA. We found instead a good positive correlation (.40) between GPA and study engagement, which is much higher than the values found by Loscalzo and Giannini (2019b) between GPA and vigor, dedication, and absorption (values ranging between .08 and .12) on Italian students.

Given the good psychometric properties of the SI-10 (with two filler items) and the sample size (*N* = 1296), which is quite big and representative of many Italian cities and many majors, we used this sample to establish the cut-off values for low and high studyholism/study engagement. Referring to the *T* scores distribution, we selected the 40th and the 60th *T* score that correspond to the following raw scores: 10 and 18 for studyholism and 11 and 18 for study engagement.

Using these values, we found that in our sample there are more engaged than disengaged studyholics, hence suggesting that it is possible to have the co-presence of a negative and a positive attitude toward study, as proposed by Loscalzo and Giannini (2017a, 2018b). Moreover, there are also more engaged studyholics than engaged students, highlighting the need for developing preventive interventions for favoring study engagement and preventing studyholism. These preventive interventions are critical, especially taking into account that in our sample the highest percentage of students belongs to the detached student category, suggesting that a lack of study engagement is spread in Italian University students.

We want to highlight that since studyholism is a new potential clinical condition, we could not perform Receiver Operating Characteristic (ROC) analysis for selecting the clinical cut-off due to the lack of a clinical sample and of clinical instruments to be used as gold standard. However, the cut-off scores we proposed allow detecting students who have sensibly higher or lower studyholism/study engagement levels than the average student. This has critical implications for the clinical practice, as the psychologist may evaluate if the student deserves an intervention addressing specifically high studyholism and/or low study engagement. In addition, by crossing the cut-off values of the two scales, he/she also evaluates if the student belongs to one of the three fewer spread types of student among the general population: disengaged studyholic, engaged studyholic, disengaged student, or engaged student. Hence, using our cut-off values helps to tailor the intervention on the student.

Finally, we used the SI-10 for analyzing some demographic and study-related differences in studyholism and study engagement, and the association between these two study-related behaviors and some academic indicators.

First, concerning *demographic variables*, we found that females have higher levels of both studyholism and study engagement than males. These results are in line with Atroszko et al. (2015); however, Loscalzo and Giannini (2019b) did not find gender-differences in study engagement. Moreover, there are no differences between students who usually do an activity (such as singing, playing an instrument or having a hobby) or a sport besides studying and students not doing such activities. Also, there is no relationship between age and studyholism or study engagement.

About *study-related variables*, the results showed that who repeated a school year before university or is currently late with his/her study has higher levels of studyholism and lower levels of study engagement as compared to who has never been rejected. We could speculate that studyholism causes academic impairment; however, future studies should evaluate this assumption by employing causal models.

Concerning differences in the year of study, we did not find any difference in studyholism. In contrast, fifth-year students have higher study engagement levels compared to the students of the previous years (but not with students in their fourth year). Keeping in mind that the SI-10 evaluates study engagement in its intrinsic motivation component, we hypothesize that students at the end of the university could be more engaged in their studies as they are nearer to their final degree. Hence, they could be more intrinsically motivated given the higher knowledge and competencies they have gained as compared to the previous years. Moreover, in Italy, the master’s degree is usually considered as more important than the bachelor degree to have a good job that matches the major of study; therefore, students could be more engaged in studying since their careers will depend on their involvement in the study.

Finally, concerning the major of study, we found that humanities and educational students (e.g., languages, literature, history, philosophy, education) have higher studyholism than both psychology and health professions (e.g., nursing) students. Moreover, the results showed that the humanities and educational group has higher levels of study engagement than the social sciences (e.g., law, economy) group. No other difference with the other two groups (i.e., medical studies and math, physics, science, and engineering) emerged. It is interesting to note that the humanities and educational group is characterized by a high level of both studyholism and study engagement. This result gives again support to the model of Loscalzo and Giannini (2017a), and more specifically to the existence of engaged studyholics, which could have some differences compared to disengaged studyholics concerning some variables. Finally, social sciences students have the lowest mean level of study engagement. This finding could suggest that who studies this major should receive an intervention aiming to detect the reason/s for the low engagement in vigor, dedication, absorption, and intrinsic motivation and target it/them to increase their study engagement.

The last analyses we performed are about the correlations between studyholism and study engagement and some *academic indicators*. We found that the two SI-10 scales are both positively and statistically significantly correlated with time spent studying, as assessed by self-reported hours per day and days per week of studying (generally and before exams). However, the values of correlation are higher for the Study Engagement scale, even if low anyway. The results we found for the Studyholism scale are a bit smaller as compared to the values found by Atroszko et al. (2015) for the correlation between study addiction and hours of study per week in total, at the university and at home (values ranging between .15 and .30, while ours range between .10 and .18). Instead, the values about study engagement are in line with Loscalzo and Giannini (2019b), whose study showed a positive correlation between the UWES-S-9 absorption subscale and the hours of study per day both in general and before exams. However, Loscalzo and Giannini (2019b) did not find a positive correlation between time spent studying and the UWES-S-9 vigor and dedication scales (the last one, being weakly and positively correlated with the hours of study per day generally, but not with the time spent studying before exams). Taken these results altogether, we suggest that the highest correlations in Italian students seem to be those between absorption (UWES-S-9 scale), inner motivation toward study (SI-10 Study Engagement scale), and time spent studying both in general and before exams. It seems evident that the more time a student spent studying and the more he/she is absorbed in studying. In the same vein, the more one is intrinsically motivated in his/her studies, the more time he/she will spend studying to get good grades. Moreover, we also speculate that the higher correlations between study addiction and time spent studying (as compared to the ones we found between studyholism and time spent studying) may be due to the less definite distinction between study addiction and study engagement – especially in its absorption component – that has been suggested for the BStAS (Loscalzo & Giannini, 2018a). In sum, these results show that preventive interventions should favor study engagement, and especially intrinsic motivation toward study, to favor academic success.

### Limitations and Strengths of the Study

About the limitations of this study, we have assessed academic performance utilizing self-reported GPA, while it would have been better to have an objective indicator. Also, the participants are predominantly females; however, this is in line with the sample used by Atroszko et al. (2015). Moreover, we analyzed some correlations and group-differences that allows making some hypotheses about the negative impact of studyholism, but that do not allow making casual assumptions. Future studies should use causal models to test the causal relationships between the antecedents and outcomes of studyholism and study engagement proposed by Loscalzo and Giannini (2017a). One first attempt in this direction has recently been made by Loscalzo and Giannini (2019a) through their path analysis model.

Besides these limitations, the SI-10 has the merit to have been created based on the suggestions of Quinones and Griffiths (2015), Kardefelt-Winther (2015), and Billieux et al. (2015) for the studying of new potential behavioral addictions (and, hence, of other new potential clinical conditions). The pool of items has been developed based on the comprehensive theory of Loscalzo and Giannini (2017a, 2018b) and with the aim of not overpathologizing a common behavior such as studying. Finally, we proposed the cut-off scores for defining high and low studyholism/study engagement and the four types of student (i.e., disengaged studyholics, engaged studyholics, engaged students, detached students).

### Conclusions

The SI-10 is a 10-item instrument with good psychometric properties. Through five items per each factor (one of which is a filler item and is not included in the scoring), it allows evaluating studyholism (or obsession toward study) and study engagement (in its intrinsic motivation component). It also allows detecting students with high/low levels of studyholism and study engagement, as well as students belonging to the following four types: disengaged studyholics, engaged studyholics, engaged students, detached students.

The SI-10 could be used in future research to understand better the features of studyholism, as well as its antecedents and outcomes. Moreover, it could be used for both preventive and clinical interventions intended to improve students’ well-being and their academic performance. More specifically, professional figures working with students (such as psychologists, counselors, and student affairs educators) may use the SI-10 in their assessment procedures to evaluate if the students they encounter in their practice and who face academic issues (e.g., low grades, dropout intention, test anxiety) have high levels of studyholism and/or low levels of study engagement. Since it is a rapid screening instrument and is straightforward to be scored, it may also be self-administered easily to all the students aiming to detect the ones in need of an intervention specifically related to studyholism and/or study engagement. Finally, the SI-10 may be administered during the implementation of interventions to increas students’ well-being and academic success to evaluate if their studyholism is decreased and/or their study engagement is increased.

## Appendix

**Table A1:** Studyholism Inventory (SI-10; English Translation of the Items)

SI-10 Item	Scoring
1. I have study-related worries even when I am not studying (e.g., on holidays, when I am doing sports)	Studyholism
2. I am satisfied with who I am as a student	Study Engagement - Filler
3. I cannot relax because of worries about studying	Studyholism
4. Often, I feel anxious or nervous because of study-related issues	Studyholism
5. The quality of my studying is a source of pride for me	Study Engagement
6. Before exams, I can’t think about anything else other than preparing for the exams	Studyholism - Filler
7. I feel overloaded with all the things I have to do	Studyholism
8. I study very hard in order to get the best grades	Study Engagement
9. My desire to get good grades motivates me to study	Study Engagement
10. I do the very best I can to get the best grades	Study Engagement

*Note.* By contacting the first author, it is possible to get the Italian, English, Polish, Croatian, and Spanish versions of the SI-10 that also includes the questions of the head-sheet.
